# Staying at work with back pain: patients' experiences of work-related help received from GPs and other clinicians. A qualitative study

**DOI:** 10.1186/1471-2474-11-190

**Published:** 2010-08-27

**Authors:** Carol Coole, Paul J Watson, Avril Drummond

**Affiliations:** 1 Division of Rehabilitation and Ageing, School of Community Health Sciences, University of Nottingham, Queen's Medical Centre, Nottingham, UK; 2 Department of Health Sciences, Academic Unit, University of Leicester, Leicester, UK

## Abstract

**Background:**

Low back pain commonly affects work ability, but little is known about the work-related help and advice that patients receive from GPs and other clinicians. The purpose of this study was to explore the experiences of employed people with back pain and their perceptions of how GPs and other clinicians have addressed their work difficulties.

**Methods:**

A qualitative approach with thematic analysis was used. Individual interviews were carried out with twenty-five employed patients who had been referred for back pain rehabilitation. All had expressed concern about their ability to work due to low back pain.

**Results:**

The perception of the participants was that GPs and other clinicians had provided little or no work-focused guidance and support and rarely communicated with employers. Sickness certification was the main method that GPs used to manage participants' work problems. Few had received assistance with temporary modifications and many participants had remained in work despite the advice they had received. There was little expectation of what GPs and other clinicians could offer to address work issues.

**Conclusions:**

These findings question the ability of GPs and other clinicians to provide work-focused support and advice to patients with low back pain. Future research is recommended to explore how the workplace problems of patients can be best addressed by health professionals.

## Background

Low back pain has considerable economic impact due to the costs associated with healthcare, sickness absence and work disability. In the UK, in the year 2008/9, an estimated 3.5 million working days were lost through musculoskeletal disorders mainly affecting the back [1]. The conclusion of a wide body of research is that low back pain should be manageable within the workplace and guidelines are available to advise GPs, other clinicians and employers on how to address the needs of workers with back pain [2-4]. Compared with most other countries within the European Union and North America there is little occupational health provision in the UK, and for the majority of the UK population the general practitioner (GP) remains the main source of advice on work-related health and sickness certification. In addition, other clinicians, particularly physiotherapists, chiropractors and osteopaths, are commonly accessed by those seeking help with back pain [5,6]. Practice guidelines recommend that all healthcare providers reassure patients with low back pain, allay fears and encourage the maintenance of, or return to, normal activity including work.

However, previous studies have identified the problems GPs have in managing consultations and sickness certification for back pain, including for example the balance of maintaining the doctor-patient relationship whilst challenging patients' expectations and perceptions of the consultation [7,8]. Other studies have demonstrated that the attitudes and beliefs of physical therapists and GPs, including their own anxieties about pain causation, influence the advice and help they give patients about work [9-11]. In contrast, few studies have examined this area from the perspective of the client. This study explores the experiences of employed people with back pain regarding the help they have received from GPs and other clinicians regarding work.

## Methods

A qualitative approach using thematic analysis was chosen [12]. The methodology has been described fully elsewhere [13] but in summary, data was collected through individual semi-structured interviews with a convenience sample of back pain patients who had been offered a seven-week community-based group rehabilitation programme (21 hours in total). The main objective of the interview was to facilitate participants to report their experiences of remaining in work with back pain. Ethical approval was granted by the Nottingham 1 Research Ethics Committee.

Participants were recruited by clinicians during routine back clinic assessment, following referral by their GP or other healthcare professional. Those eligible were employed, concerned about their ability to work due to back pain, fluent in English and had been offered group rehabilitation.

The interviews took place during July and August 2008, either at the participant's home, workplace, or at a local clinic. Interviews lasted approximately 45 minutes and were digitally recorded. Written consent was gained at the interview.

A list of topic areas using open questions and prompts was developed through a review of the literature and by discussion, with two user representatives, and between the authors. Topics included the effect of back pain on work ability; the help received from clinicians in managing at work; expectations of rehabilitation regarding work.

The interviews were conducted and recorded by one of the researchers (CC) and transcribed verbatim. To manage the data systematically NVivo8, (QSR International PTY Ltd) a qualitative software package, was used to help code each transcript; initial codes were refined following constant comparison of the scripts. As the data collection proceeded, themes were identified and analysed by repeated study of the scripts and discussion with the research team. When all the data had been collected and coded, two of the researchers (CC and PJW) reviewed and agreed the final themes.

## Results

Twenty-five patients participated in the study, representing private and public sector workers; professional, skilled, semi-skilled and unskilled work; manual and non-manual occupations. Twenty worked for large employers (> 250 employees). Six had never taken sick leave for back pain. Six were off sick due to back pain at the time of the interview, two for more than six months. The mean age of the participants was 44.7 years (range 22-58 yrs), with a mean back pain history of 6.8 years. Twelve were male, thirteen female. Further demographic details have been published previously [13].

Most, but not all of the participants had been referred to rehabilitation by their GP. In some cases this was as a result of seeing a different GP at the practice than usual, or had arisen during a consultation about another condition. A few had been referred from secondary care, e.g. Rheumatology or Pain Clinic.

The main finding from this study was that the participants reported receiving little appropriate work-related help from health professionals. Five themes were identified through analysis of the scripts which best characterized their experiences. Each theme is illustrated in the text with quotations from the participants.

### 1. Doubts as to what GPs have to offer those working with back pain

There was little evidence that the participants expected GPs to offer them advice about work. Many perceived that there was little to be gained by consulting their GP about back pain. Some had sought private investigations or physical therapy instead. They saw the main role of the GP as prescribing medication and providing sickness certificates. This participant described how she had previously managed a long history of recurrent back pain:

I didn't go to the doctors much with it because I thought, it's a bad back, you know, there's no point. (Female interviewee 24 age 51)

A 22 year old participant reported that she had not received any advice about work from her GP, and had delayed consulting him previously as a result of advice from family and friends. She had changed to a job that would accommodate her back pain:

When I first hurt my back everybody said 'don't bother going to your doctor about it, they can't do anything, just rest up a bit'. (Female interviewee 27)

Another believed others were better placed to manage back pain and had sought a consultation and investigations through private healthcare:

I don't want to be critical of my GP but my understanding is they're not the right people to deal with back problems. I don't mean that disrespectfully, I mean that because it has to be passed on somewhere. (Male interviewee 23 age 57)

Participants varied in their relationship with their GP. The frequency with which patients had consulted their GP is not known. Some had rarely needed to consult their GP or had chosen not to as a result of previous experiences. Some reported a very good relationship, others less so. Several reported different experiences within the same practice or by changing practices. Few expressed dissatisfaction with their GP, but there was a general acceptance that GPs could offer little in the way of help.

### 2. Little evidence of effective advice about work from the GP

When they had consulted their GP, many participants reported that they had not received any advice or support in relation to work that they had found effective. It seemed that some GPs were more inclined to offer help than others. One participant who worked for a large public employer described how her GP had not considered it to be his role:

I went in, he was a young doctor, he says 'You work for xxxx.? Why haven't you been to Occupational Health? We haven't got time to deal with things like this. It's not up to us, you should have been to Occupational Health'. (Female interviewee 24 age 51)

An office worker reported that his back pain had been ignored by his manager. His GP's response was to encourage him to stay at work, but in the absence of tailoring this advice to the workplace the participant felt that this simply demonstrated a lack of appreciation of his condition. He reported how he had been able to remain in work because he had a part-time job:

GP - he just said if you could stick at your job it's better for you. To keep working as much as you can. Keeping active. I think I learnt later that they have no idea about back problems........I haven't been off sick with my back - because I do part-time anyway so I try and take it easy during the day, and then I can keep at work. (Male interviewee 7 age 43)

Another example of advice out of context of the workplace is offered by this self-employed participant:

I rang the GP and said 'Look, I don't know what's happened - I think I've done my back in. I've never had backache or a back injury before - what should I do? Should I go to A & E?' He says 'Nothing, have a paracetamol'. For six and a half months I didn't do any work at all. (Male interviewee 2 age 43)

As a result, this participant reported that he then delayed consulting his GP further because he was upset at the GP's response. He had continued to self-manage, working at a reduced rate for more than two years until eventually referred for rehabilitation.

### 3. GP and clinician management may increase concerns about working with back pain

Several participants described how GPs and other clinicians advised avoidance of work or particular tasks, implying that work would exacerbate their condition or could place them at risk, rather than form an essential part of their recovery.

What did the chiropractor say about work? *No. He said no. Don't go back. Because I don't think he really understood what I did. (Female interviewee 26 age 51)*

Another participant describes work-related advice from her physiotherapist:

She said I would be OK to go back to work, but don't do any heavy lifting .......after I've completed the programme - by then I should be OK, and I will have learnt ways to deal with lifting. (Female interviewee 21 age 41)

It seemed that rather than making contact with the employer and advising on temporary modifications, clinicians gave generally vague and negative advice, such as 'take it steady' and 'be careful' or implied that work was harmful, even in this case where the physiotherapist was based at the workplace:

*The first time I went to see him *(physiotherapist) *he said 'D'you think you ought to be here'? (at work) - and I said 'Yeh, why?' And he says 'Well, what about the pace?'. And I said 'Well what about it?' I mean, I think I've got a pretty high tolerance of pain anyway. It's probably the age I am - old school sort of thing! (Male interviewee 8 age 53)*

Those in manual work were more likely to receive warnings:

He (GP) asked me what job I did, and I told him. And he says 'Have you looked for a job that's lighter work?' And as I says to him 'you do your job because you enjoy it. If I wanted a job doing light work I would have found one a long time ago'. (Male interviewee 22 age 35)

### 4. GPs are more inclined to write sickness certificates than help patients manage work problems

It seemed that GPs were more inclined to provide sickness certification than interventions aimed at work retention. This building worker describes refusing the offer:

*It's the same old scenario. He said take time off. He could have quite happily wrote me a note off (sickness certificate). I don't know how long he intended me to have off, but it's a tight budget this house, because there's only actually me in the house earning money*.

Nevertheless, remaining at work had an impact on other aspects of his life:

I just keep going. I just deal with it at the weekends. (Male interviewee 20 age 43)

Another participant described how he had to take the initiative in requesting that his GP recommend him for modified duties (this was the only example given of a GP using the 'remarks' section on the current sickness certificate which can be used to advise the employer regarding work tasks):

I went to the doctor and he wanted to put me off sick then but I said 'No I want to stay at work' and I told him to give me a note which would put me on light duties for four weeks where I refrain from any heavy duties and I'd take it to my boss, and I said to the doctor 'They'll be all right with that, I'll take it in'. He said 'Yeh, do what you can'. (Male interviewee 3 age 44)

Some felt they had to comply with their GP's wishes. For this participant, lengthy certification had become a routine method of management:

*He normally gives me a paper *(sickness certificate) *for about four weeks and then I have to go back and see him - and then he's put me on another four weeks because he wants me to see the Pain Management and the back team before I went back to work. Which I have done, so I'll just have to - hopefully he'll let me back next week. (Female interviewee 11 age 57)*.

Others had been signed off work by the GP while waiting for the results of tests and investigations:

*He signed me off for two weeks at first and said 'let's wait till we get the results of the MRI' and when the results came he said 'I'm going to refer you to the back team' and he sent me a paper *(sickness certificate) *for six weeks. (Female interviewee 17 age 37)*

### 5. Lack of dialogue between GPs, clinicians and employers

There was little evidence of dialogue between GPs and other clinicians and employers, leaving the participants responsible for channelling and interpreting information between the two sectors. This could leave them with concerns as to whether their employers would believe their condition was valid:

Sometimes I wish they would do - my doctor and employers would get in touch with each other because - maybe it's just me, but I think when I ring up work, you know, I feel sometimes - I bet they don't believe me. (Female interviewee 11 age 57)

In only one case had a GP contacted a participant's employer about the management of her back pain at work. This was in writing and the participant reported it had no impact. Two participants reported that therapists had written letters for them to give to their employers recommending alterations to workstations, but there was no direct contact, and their action had not fully resolved the problem:

He wrote a letter. I showed that to the manager. Things have improved slightly - they've been out and bought me a new chair. It's not ideal, but it's better than the one I had. I did want to have a separate monitor with a keyboard raised up and - but that hasn't come to fruition yet. I'm still waiting on that. (Female interviewee 25 age 46)

For this participant, therapists had been successful in helping her get a more comfortable chair, but underlying organisational barriers were not addressed:

*The problem is, the physio recommended that every half an hour I have a break, and the computer programme gets turned off. But that can't happen in my job because of the nature of it - I can't turn my computer off. So that part I couldn't actually instigate. (Female interviewee 12 age 30)*.

Most were generally in favour of contact being made between healthcare practitioners and the workplace. This was a typical response when asked their opinion as to whether they thought the rehabilitation team might have any contact with their employer:

Wouldn't bother me - if they felt there was something that they could do to help things then - by all means do it. (Female interviewee 25 age 46)

And this participant felt that contact between physiotherapy and his employer may have helped him to retain his previous job:

It probably would have been nice just to have a bit more communication. Whether he would have acted on it or not I don't know. But if it's coming from somebody else as an outsider saying 'look we're monitoring him and this is what's up, he's got to go on lighter duties. Or have an assistant or something like that for the real heavy work, just to help, and he is on the mend, blah blah blah', he's probably look at it in a different way. (Male interviewee 22 age 35)

## Discussion

This study aimed to explore patients' experiences of the help they had received from clinicians in regard to working with low back pain. We found that most had consulted other clinicians as well as their GP about their back pain but had received little work-focused guidance or support. Many had remained in work despite, rather than due to the recommendations they had received, and, moreover, there was little expectation among the participants as to what GPs and other clinicians would be able to offer to address their problems in the workplace.

Studies of back pain prevalence have demonstrated that only between 30% and 40% of those with back pain will consult a GP [14,15]. Little is known as to why people choose not to consult their GP. Our study has shown that employees may remain at work with back pain without visiting the GP believing that GPs have little to offer. We have demonstrated that workers may alter their hours, duties or their job to accommodate their pain; they may limit their career options; working in pain may impact on their lives outside work. These changes appear to be self-imposed without recourse to evidence-based support and help. People may remain at work whilst feeling unwell, termed 'presenteeism' which may have a detrimental effect on their future health, their work performance and productivity [16]. Patients may therefore not consult their GP until the situation at work has deteriorated and is more difficult to resolve. Alternatively they may consult other healthcare professions instead, particularly for manual therapy which is recommended as a core intervention for non-specific low back pain [17]. A recent study by Pincus et al [18] suggests that low back pain comprises 70% of the caseload of private musculoskeletal practitioners, and that these tend to be patients with long term recurrent symptoms rather than acute episodes. A study by Foster et al [5] concluded that low back pain accounted for at least 50% of physiotherapists' workload; this proportion may increase further as the government intends to increase the provision of self-referral to NHS physiotherapy [19].

There is a wealth of evidence that temporary modifications can aid work retention [20]. Few participants in our study reported being assisted or advised on modified work, and those who did described it as vague and not fully integrated into the workplace. Simply advising a patient to stay at work, although reflecting clinical guidelines to remain active, is of little practical help and may be misconstrued by patients as a lack of understanding of what it means to remain at their workplace with back pain. Sickness certification was the main way in which GPs managed difficulties at work, even in workers who expressed a willingness to remain at work. This supports recent studies which have shown that patients' feel doctors are too busy, reluctant or unable to address work-related issues [7,21,22].

In the UK, the existing sick note is to be replaced by a 'fit note' whereby GPs will be expected to offer a greater depth and range of advice on fitness for work and work modifications [19]. The ability and willingness of GPs to make effective use of the new fit note has been questioned generally [23] and more specifically with this client group [24]. Early return to work with a musculoskeletal disorder has been associated with GPs providing advice on managing a recurrence and contact with the workplace [25] but these are not requirements of the fit note. The 'work-focused' advice and support provided by healthcare providers will be dependent on their knowledge, skills, attitudes and beliefs. Further training may help to address educational needs, but may be insufficient alone in addressing attitudes; having an interest in back pain does not necessarily improve occupational management [26] and a recent systematic review has concluded that there is inconsistent evidence that educating doctors in evidence-based guidelines has a positive effect on their management of low back pain [27]. Participants in this study had received care from both private and public health providers but the lack of appropriate or effective work advice remained constant. Some authors have suggested fear-avoidance beliefs of GPs and other clinicians are a factor: those who perceive low back pain as mainly a biomechanical condition are more likely to advise people to refrain from work or avoid certain tasks [9,11]. This is seen in public and private practitioners [18] and our participants' experiences support these findings.

One participant gave an example of a GP making a direct attempt to influence the employer and in two other cases, therapists had tried to improve patients' workstations, but all reported limited success. Unfortunately a patient's employer is under no obligation to act on advice given. The roles and responsibilities of healthcare professionals in relation to their patients'employment are poorly defined in the UK. GPs may expect patients to receive help from occupational health, but few UK employees have access to these services [28]. Physical therapists may expect workplace assessments and modifications to be the role of health and safety officers or occupational therapists [18]. Laypersons/patients on the other hand, may see themselves as responsible for managing musculoskeletal disorders [29] and/or have varied expectations of the help that GPs and clinicians can provide. UK healthcare professional bodies have signed a Consensus Statement, pledging to 'do all we can to help people enter, stay in or return to work' [30], but as yet, with no clear lines of responsibility or pathways of communication, patients seem to be left to rely on their own resources.

### Strengths and limitations of the study

Although semi-structured interviews based on previously published research gave a sound theoretical backing to the research, they may have constrained the early interviews. The interview guide was revised as the interviews progressed in order to explore some of the topics in greater depth. Dependability was increased by having the same interviewer who transcribed the interviews verbatim. Interview transcriptions and suggested themes were repeatedly checked, compared and revised with one of the authors (PJW) in order to increase credibility and dependability, although in this study they were not confirmed by the participants.

The method of convenience sampling was chosen because of restraints on time and resources and this restricts the generalisability to all people working with low back pain. There were comparatively few participants self-employed or employed by small-to-medium enterprises compared with large employers. The reason for this is unclear. It may be that the pressures of working for oneself or for a small employer impose actual or perceived obstacles to accessing treatments or taking part in a research study.

A strength of the research is that it provides a broad understanding of the issues involved, although the diversity of the sample also limits the potential to understand in depth/distinguish between the experiences of different sub-groups in terms of for example age, gender, occupation.

Other factors are essential to consider in the overall study of work retention and with low back pain such as the context of the workplace and home situation, but were not the aim of this piece of research. However, our study does highlight the complexity of issues and challenges specific to the context of healthcare.

## Conclusion

There was little evidence in our study that GPs and other clinicians were effectively managing the work issues of their patients with back pain. The participants appeared to be mostly managing these issues themselves or receiving inadequate or inappropriate advice. Our results suggest that we should question the role of the GP as manager of sickness certification and the ability and attitudes of clinicians generally to provide work-focused support. Recent guidance on the roles of employers and clinicians has been published [4] but further research is needed to explore when, how and by what means GPs and clinicians can best apply this guidance within the UK's work and healthcare context. Patients' expectations of healthcare regarding work support and advice may need to be challenged and services made more accessible to employers and employees. Relying on the GP or other clinicians to provide work-focused support and advice to patients with low back pain is unlikely to be sufficient.

## Competing interests

The authors declare that they have no competing interests

## Authors' contributions

CC conducted and transcribed the interviews and drafted the manuscript. CC conducted the initial data coding and analysis. PJW independently checked and reviewed the coding and analysis. CC and PJW identified and reviewed the themes. PJW and AD participated in revising the manuscript. All authors read and approved the final manuscript.

## Pre-publication history

The pre-publication history for this paper can be accessed here:

http://www.biomedcentral.com/1471-2474/11/190/prepub
